# Functional Porous Carboxymethyl Cellulose/Cellulose Acetate Composite Microspheres: Preparation, Characterization, and Application in the Effective Removal of HCN from Cigarette Smoke

**DOI:** 10.3390/polym11010181

**Published:** 2019-01-21

**Authors:** Peijian Sun, Song Yang, Xuehui Sun, Yipeng Wang, Lining Pan, Hongbo Wang, Xiaoyu Wang, Jizhao Guo, Cong Nie

**Affiliations:** Zhengzhou Tobacco Research Institute of CNTC (China National Tobacco Corporation), Zhengzhou 450001, China; yangsong@mail.ustc.edu.cn (S.Y.); xuehui_sun1234@aliyun.com (X.S.); yipeng_w@sina.com (Y.W.); panlining@126.com (L.P.); whbhn@126.com (H.W.); wangxiaoyu@iccas.ac.cn (X.W.); guojizhao@126.com (J.G.)

**Keywords:** porous microspheres, carboxymethyl cellulose, cellulose acetate, cupric ions, chemisorption, hydrogen cyanide, smoke yield

## Abstract

To selectively reduce the yield of hydrogen cyanide (HCN) in the cigarette smoke, functional porous carboxymethyl cellulose/cellulose acetate (CMC/CA) composite microspheres were prepared via the double emulsion-solvent evaporation method. Cupric ions, which have a high complexing ability toward HCN, were introduced to the CMC/CA composite microspheres during the fabrication process via an in situ ion cross-link method. The microspheres were characterized using nitrogen adsorption, mercury intrusion porosimetry, and scanning electron microscopy (SEM). The microspheres have a predominantly macroporous structure indicating weak physisorption properties, but sufficient functional cupric ion groups to selectively adsorb HCN. With these CMC/CA microspheres as filter additives, the smoke yield of HCN could be reduced up to 50%, indicating the great potential of these microspheres as absorbents for removing HCN from cigarette smoke.

## 1. Introduction

Cigarette smoke, which is well-recognized as one of the most important causes of lung cancer, chronic obstructive pulmonary disease, and cardiovascular disease is an extremely complex aerosol stream that consisted of more than 5000 identified chemicals, covering a range of chemical groups and functionalities, as well as a board range of volatilities, from permanent gases to compounds with low vapor pressures. Amongst the identified chemicals in cigarette smoke, approximately 150 constituents have been documented as toxicants [1], in which, hydrogen cyanide (HCN) has been assigned as one of the 18 priority toxicants in cigarette smoke for reporting and regulation [2,3]. HCN could inhibit cytochrome oxidase in the respiratory chain and thus has a very high toxicity. The intake of HCN with an oral dose of 0.5–3.5 mg/kg body weight or inhalation of 270 mg/L of air for only a few minutes could cause immediate death in humans. As for smokers, the chronic exposure to hydrogen cyanide could lead consequently to amblyopy, retrobulbar neuritis, and sterility, and is involved in impaired wound healing [2]. Thus, the reduction of the toxicant yield in cigarette smoke, such as the smoke HCN yield, is important for both environment protection and public health.

From the 1950s onwards, many attempts have been made to selectively reduce toxic constituents from the cigarette smoke. The most-used technique to remove some of the volatile toxicants from cigarette smoke is adsorption by varied porous adsorbents [4,5,6,7,8]. Active carbon, which is the most used adsorbent in cigarette filters, could reduce a broad range of volatile smoke constituents by the non-selective physisorption between the adsorbents and the smoke constituents [4]. However, as for the absorption of permanent gases and smoke toxicants with high vapor pressures, such as HCN, formaldehyde, etc., the physisorption showed a less-effective performance [5]. As for adsorption in cigarette smoke, adsorbents need to operate under a very high flow rate at the gas–solid interface in the presence of thousands of other chemicals that cover a range of chemical groups and functionalities. The flow rate of the cigarette smoke is as high as 17.5 mL per second for a typical machine–smoke regime (ISO 3308:2012), and therefore the contact time of the adsorbents and the smoke toxicants is very short. In addition, the extremely complex constituents in the smoke with varied functionalities also made the adsorption act difficult and challenging. Thus, the physisorption capacity could be greatly reduced or even lost in the extremely complex aerosol ambient. The modification of the adsorbents by introducing functional groups was developed to improve the adsorption capacity and reduction efficiency of smoke toxicants that have high vapor pressures at ambient temperature. For instance, the modification of active carbon by impregnation with CuO showed an improved adsorption capacity of HCN using a chemisorption mechanism and has been used in personal protective equipment [6]. The modification of an ion-exchange resin by introducing functional amino groups also showed an enhanced reduction of HCN and aldehydes from the cigarette smoke [7]. Thus, chemisorption may therefore represent a feasible mechanism to selectively reduce HCN from the cigarette smoke.

One of the main requirements for the adsorbents is a low cost/benefit ratio. Cellulose, constituting the most abundant polymer resource, is one of the low-cost materials. However, cellulose is not soluble in water and most organic solvents, and it had a poor reactivity, which makes it difficult to be directly modified to fabricate other useful materials [9]. An effective means to cope with the dilemma is the modification of cellulose by grafting the cellulose with functional polymers containing abundant functional groups, such as carboxyl, amino, and amide groups [10,11,12]. These adsorbents usually exhibit high adsorption capacity and high selectivity. However, the residues of unreacted initiators and monomers usually have significant toxicities, which would lead to serious environmental pollution. Another way to overcome these drawbacks is by using the commercially available cellulose derivatives, such as cellulose acetate (CA) and carboxymethyl cellulose (CMC). Among the cellulose derivatives, cellulose acetate (CA) is an important cellulose ester in industry due to its desirable physical properties. CA is widely used as a filter material in cigarettes and as a film base in photography [13,14]. The modification of commercially available CA could also enable cellulose derivatives for other functions. For instance, by introducing functional groups, such as carboxyl and amino groups, CA could bond with heavy metal ions through surface complexation mechanisms [15,16]. Carboxymethyl cellulose (CMC) is another important derivative of the cellulose family with the carboxymethyl groups bound to some of the hydroxyl groups on a cellulose backbone, which is usually made via the alkali-catalyzed reaction of natural cellulose with chlorinated acetic acid [11]. The polar carboxyl groups render the cellulose soluble and chemically reactive. CMC is widely applied in a lot of industrial sectors including food, paper making, paints, pharmaceutics, cosmetics, and water treatment [17,18,19,20,21,22,23]. The polar carboxyl groups would also enable the complexion of functional heavy metal ions onto the cellulose derivatives to provide an additional chemisorption of HCN to the CMC backbone. CMC and CA, the two most-used commercially available cellulose derivatives, were therefore chosen to fabricate into porous composite microspheres to reduce HCN from cigarette smoke. The CMC was functionalized with cupric ions to act as active sites for chemisorption of HCN via a complexation mechanism. CA was chosen due to its wide usage in cigarette filter materials and the easy fabrication into porous microspheres with connected pores, which is beneficial for the smoke passing through and contacting with the active sites. Combining these benefits, a high HCN removal efficiency is expected for these porous composite microspheres.

In this study, porous CMC/CA composite microspheres were prepared via the double emulsion-solvent evaporation method. Cupric ions, which have a high complexing ability to HCN, were introduced to the CMC/CA composite microspheres during the fabrication process via an in situ ion cross-link method. The influence of preparation parameters on the microstructure of the resultant CMC/CA microspheres was investigated. Finally, the removal of HCN from cigarette smoke by using these functional microspheres as absorbents was investigated.

## 2. Materials and Methods 

### 2.1. Materials

Cellulose acetate (CA) with an acetyl content of 39.8 wt % was supplied by Eastman Chemical Company (Shanghai, China). Carboxymethyl cellulose (CMC) with a degree of substitution (DS) of 0.7 and a viscosity of 50–100 mPa·s, and polyvinyl alcohol (PVA, Type 1788) were supplied by Shanghai Aladdin Bio-Chem Technology Co., Ltd., Shanghai, China. Other reagents were of analytical grade and used without further purification.

### 2.2. Preparation of CMC/CA Composite Microspheres

**Figure 1:**
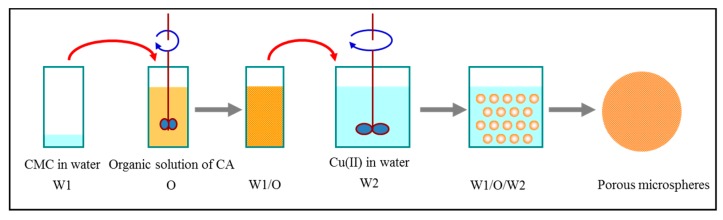
Synthesis pathway of CMC/CA microspheres.

Porous CMC/CA composite microspheres were prepared using the double emulsion-solvent evaporation method, and the cupric (Cu^2+^) ions were introduced to the CMC/CA composite microspheres during the fabrication process via an in situ ion cross-link method (Figure 1). Typically, 50 mL of the inner aqueous solution (W1) with a 3 wt % of CMC was added into 100 mL of 5 wt % CA solution in a mixture of ethyl acetate/dichloromethane (50 wt %) to prepare the primary emulsion (W1/O). This primary emulsion was emulsified using a homogenizer (T25, IKA, Staufen, Germany) at 10,000 rpm, and then added into 800 mL of 0.2 wt % PVA solution (W2) containing 50 mmol/L cupric sulfate under stirring to form the secondary emulsion (W1/O/W2). Stirring was continued for at least 4 h to allow the evaporation of the solvent. The produced CMC/CA microspheres were collected and thoroughly washed with deionized water and then ethanol. The microspheres were then separated using standard sieves and dried under 60 °C.

### 2.3. Characterization of Microspheres

Fourier transform infrared (FTIR) analysis was performed with a NEXUS-470 (Thermo Nicolet, Madison, WI, USA) FTIR spectrophotometer, taking 32 scans for each sample. The surface and internal structure of the microspheres were characterized with an Inspect F50 (FEI, Hillsboro, OR, USA) scanning electron microscope (SEM). The samples were mounted directly onto the SEM sample holder using double-sided sticking tape and were sputter-coated with gold in a vacuum prior to measurements. For internal structure characterization, the microspheres were cut into halves in liquid nitrogen and then subjected to SEM for their internal morphology inspection. The elemental analysis of the microspheres was determined using an INCA X-MAX (Oxford Instruments, Abingdon, UK) energy dispersive X-ray spectrometry (EDS) after the SEM procedure. The X-ray spectrum was conducted with 10 keV in EDS procedure. The X-ray spectrum was then used for semi-quantitative analysis through the elemental percentage mode of the standard less approach. Nitrogen adsorption measurements were performed on a Tristar II 3020 (Micromeritics, Norcross, GA, USA) gas sorptometer with the sample maintained at −196 °C using liquid nitrogen. Samples were degassed at 80 °C for 10 h under vacuum prior to isotherm determination. The surface area was calculated by the standard Brunauer-Emmet-Teller (BET) method. Mercury intrusion porosimetry was performed on an Autopore IV 9500 system (Micromeritics, Norcross, GA, USA). The loading amounts of Cu^2+^ were measured using a 7500 (Agilent, Santa Clara, CA, USA) inductively coupled plasma mass spectrometry (ICP-MS), following the extraction of Cu^2+^ into an aqueous solution by immersing 20–40 mg of microspheres in 1000 mL of 3 wt % nitric acid for 4 h.

### 2.4. Adsorption of HCN from Cigarette Smoke

**Figure 2:**
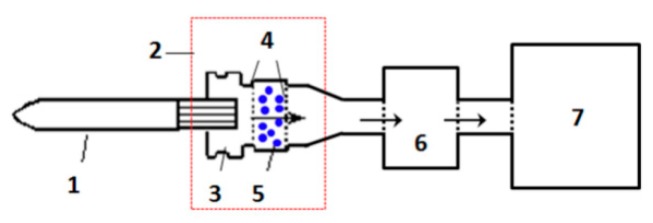
Device to simulate the addition of absorbents to the cigarette filter. (1) cigarette, (2) homemade device, (3) cigarette holder, (4) 120 mesh sieve, (5) sample, (6) Cambridge filter pad, and (7) smoking machine.

The adsorption of HCN from cigarette smoke using these functional microspheres was performed on a homemade device (Figure 2). The added amounts of samples were 15 mg for a cigarette. The test cigarettes had a circumference of 24.1 mm and were made up of a 59 mm long tobacco rod containing a Virginia style tobacco (from Henan province, China) and a 25 mm length unventilated CA filter. Mainstream smoke yield was measured using the standard ISO smoking regime with a puff volume of 35 mL, puff duration of 2 s, and puff interval of 60 second on a SM-450 linear smoking machine (Cerulean, Milton Keynes, UK), according to ISO 3308:2012 (“Routine analytical cigarette-smoking machine—Definitions and standard conditions”). HCN in gas phase of the smoke was collected by passing the whole mainstream smoke stream through an impinger of sodium hydroxide solution. The smoke particulate phase was collected on a glass filter pad (referred to as a Cambridge filter pad) and extracted in sodium hydroxide solution via shaking for 30 min. HCN was quantified using a colorimetric reaction and a continuous flow analyzer system.

## 3. Results and Discussion

### 3.1. Preparation and Characterization of CMC/CA Composite Microspheres

Porous CMC/CA composite microspheres were prepared using the combination of the double emulsion-solvent evaporation and in situ ion cross-linking method (Figure 1). Cupric (Cu^2+^) ions were introduced to the CMC/CA microspheres during the in situ cross-linking process. During the microspheres formation in the double emulsion-solvent evaporation process, the inner CMC aqueous solution was cross-linked via the cupric ions which diffused from the outer aqueous solution.

**Figure 3:**
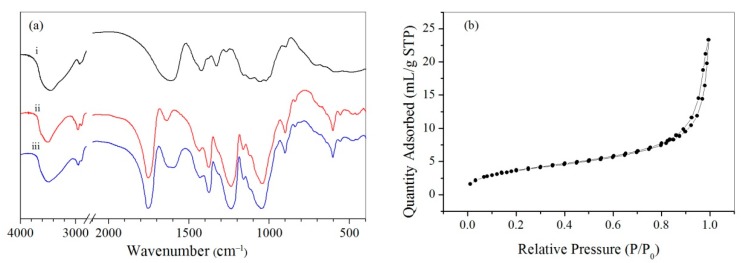
(**a**) FTIR of CMC (i), CA (ii), and CMC/CA microspheres (iii). (**b**) Typical nitrogen adsorption curves of CMC/CA microspheres.

In the FTIR spectrum (Figure 3a) of CMC/CA microspheres, the absorption at 1753 cm^−1^ was attributed to acetyl group vibrations (–C=O) of CA; the absorption at 1597 cm^−1^ was attributed to acetyl group vibrations (–C=O) of carboxyl groups that complexed to cupric ions, showing a red shift compared to CMC, which showed an absorption at 1640 cm^−1^. The red shift was due to the complexing of carboxyl groups in CMC to cupric ions [24]. Figure 3b show typical nitrogen isotherm for the microspheres at −196 °C. The isotherm was type IV, indicating a predominantly macroporous structure, which was in accordance with mercury intrusion porosimetry results. 

**Figure 4:**
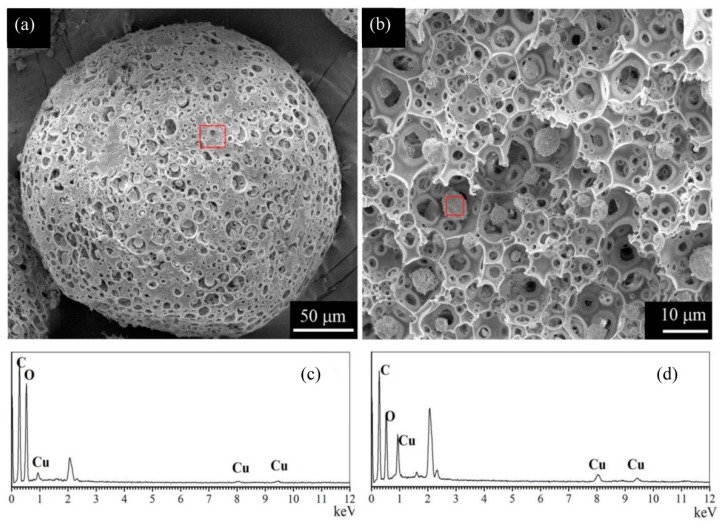
(**a**,**b**) SEM images of CMC/CA microsphere. (**c**,**d**) The corresponding EDS spectra of CMC/CA microsphere. (**a**,**c**) denotes the surface structure while (**b**,**d**) were the cross-section structure of the microspheres.

Figure 4a–d showed the SEM and EDS results of a typical CMC/CA microsphere. The composite microspheres had a macroporous structure and smaller microparticles could be observed in the caves of the composite microsphere. EDS results showing the cupric(II) content of the inner microparticles was ≈10.5%, which was much higher than the surface cupric(II) content of CMC/CA microsphere (ca. 3.1%). The inner microparticles were supposed to be the cupric cross-linked CMC.

### 3.2. Influence of Preparation Parameters on the Microsphere

To investigate the effect of different formulation and process on microspheres properties, various batches of formulation were performed by varying the CMC concentration of the inner aqueous solution, the W1/O ratio, and the Cu(II) content of the outer aqueous solution, as given in Table 1.

#### 3.2.1. CMC Concentration in the Inner Aqueous Solution

**Figure 5:**
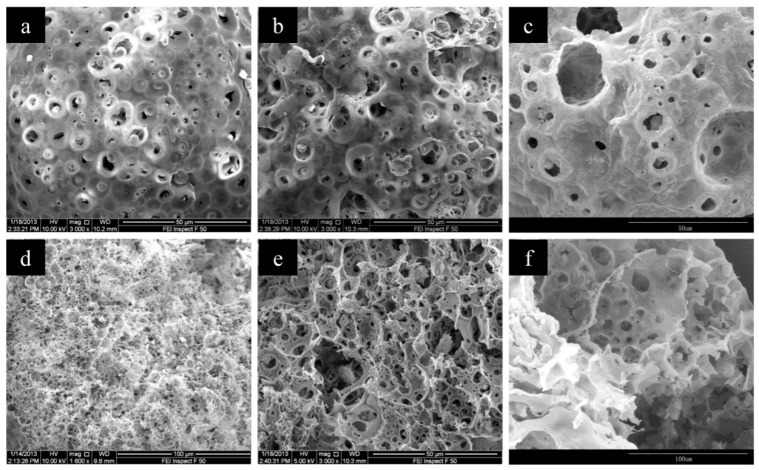
The SEM images of CMC/CA microspheres fabricated with 2% (**a**,**d**), 3% (**b**,**e**), 4% (**c**,**f**) CMC concentrations. Images (**a**–**c**) were the surface images while (**d**–**f**) were the cross-section images of CMC/CA microspheres.

**Figure 6:**
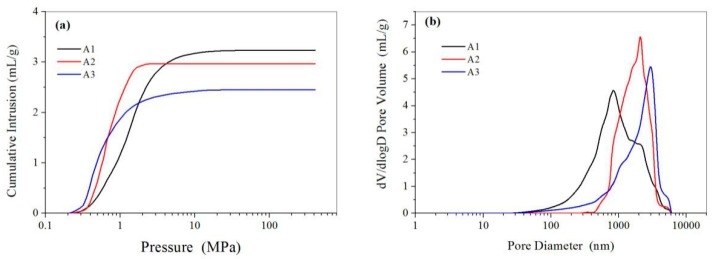
(**a**) Cumulative mercury intrusion traces and (**b**) pore size distribution of the CMC/CA microspheres fabricated with 2% (A1), 3% (A2), 4% (A3) CMC concentration.

For the fabrication of porous microspheres by the combination of emulsion–solvent evaporation and in situ ion cross-link method, CMC plays as both an emulsifier and functional component to load the cupric ions. The microspheres prepared with a CMC concentration varying from 2% to 4% all have macroporous structures, and the microsphere prepared with a higher CMC concentration (e.g., 4%) showed a larger pore size while a decreased pore number, as seen from the SEM images in Figure 5. Mercury intrusion porosimetry results showed that while the CMC concentration increased from 2% to 4%, the pore size increased from 952 to 2264 nm and the pore volume decreased from 3.23 to 2.45 mL/g, as shown in Figure 6 and Table 1. The changes of the microsphere structure were because of the increasing viscosity of a higher CMC concentration. The dispersion of the inner aqueous solution with a higher viscosity in W1/O was difficult and thus resulted in an increased pore size and a decreased pore volume of the resultant microspheres. Due to the predominantly macroporous structure, the microspheres had relatively small BET surface areas of ≈5.0–6.5 m^2^/g. The loading amount of Cu(II) for the microspheres prepared using 2%, 3%, and 4% CMC concentration was 1.8, 2.6, and 3.3 wt %, respectively. With an increased CMC concentration, more Cu(II) could be complexed to the CMC/CA microspheres.

#### 3.2.2. The W1/O Ratio

**Figure 7:**
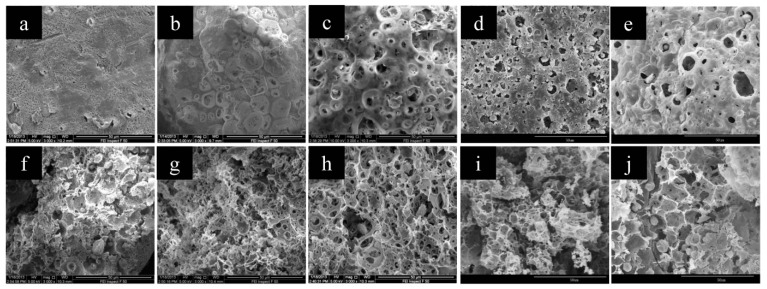
The SEM images of CMC/CA microspheres fabricated with W1/O ratios of 10/100 (**a**,**f**), 30/100 (**b**,**g**), 50/100 (**c**,**h**), 70/100 (**d**,**i**), and 90/100 (**e**,**j**). Images (**a**–**e**) were the surface images while (**f**–**j**) were the cross-section images of the CMC/CA microspheres.

**Figure 8:**
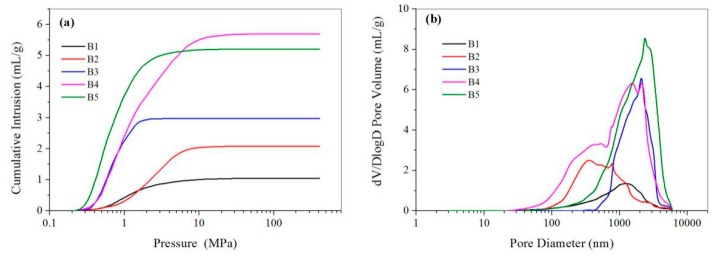
(**a**) Cumulative mercury intrusion traces and (**b**) pore size distribution of the CMC/CA microspheres fabricated with W1/O ratios of 10/100 (B1), 30/100 (B2), 50/100 (B3), 70/100 (B4), and 90/100 (B5).

The W1/O ratio had great influence on the microsphere structures. As seen from the SEM results in Figure 7, the surface structures of CMC/CA microspheres changed from a relatively smooth surface to a porous surface as the W1/O ratio increased from 10:100 to 70:100, while larger and more pores could be observed from the cross-section images of the microspheres with a higher W1/O ratio. The mercury intrusion porosimetry results showed that the pore volume increased from 1.04 to 5.69 mL/g while W1/O increased from 10:100 to 70:100, but a slight decrease of pore volume was observed for the microspheres with W1/O of 90:100 (Table 1 and Figure 8). As the W1/O ratio increased, the BET surface areas increased from 2.24 to 13.50 m^2^/g, but decreased to 7.42 m^2^/g when the ratio of W1/O increased to 90:100, which had a similar trend to the pore volume. During the microsphere fabrication process, the inner CMC aqueous solution (W1) was cross-linked by Cu^2+^ to form CMC microparticles (microgels). The collapse of the CMC microparticles during the drying process resulted in the macropore structure in the composite microspheres. With a larger W1/O ratio, the proportion of the inner aqueous solution (W1) in the primary emulsion increased, thus resulting in a larger pore volume and BET surface. However, the stability of the primary emulsion decreased with a much larger W1/O ratio of 90:100. The fusion and deformation of the inner aqueous solution in the primary emulsion resulted in a decreased pore volume and BET surface area. With the increased W1/O ratio, more cupric ions could be complexed to the resultant microspheres and thus an increased loading amount of Cu(II) was observed.

#### 3.2.3. The Cu(II) Content in the Outer Aqueous Solution

**Figure 9:**
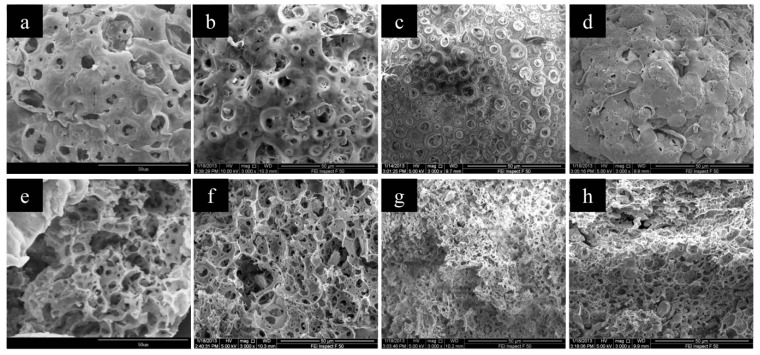
The SEM images of CMC/CA microspheres fabricated with Cu(II) content of 25mM (**a**,**e**), 50mM (**b**,**f**), 100mM (**c**,**g**), 200mM (**d**,**h**) in the outer aqueous solution. Images (**a**–**d**) are the surface images while (**e**–**h**) are the cross-section images of the CMC/CA microspheres.

**Figure 10:**
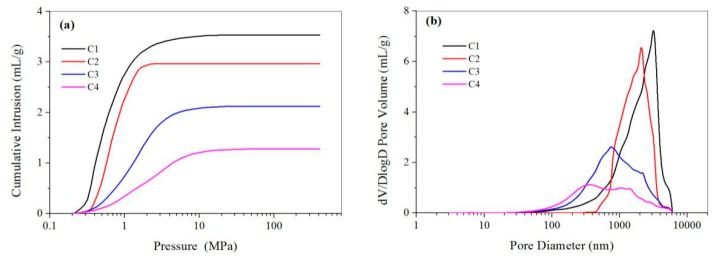
(**a**) Cumulative mercury intrusion traces and (**b**) pore size distribution of the CMC/CA microspheres fabricated with Cu(II) content of 25mM (C1), 50mM (C2), 100mM (C3), and 200mM (C4) in the outer aqueous solution.

While the Cu(II) content in the outer aqueous solution increased from 25 to 200 mM, the BET surface area decreased from 5.86 to 2.80 m^2^/g (Table 1). SEM results showed the microspheres have a relatively smooth and compact surface when fabricated with a higher Cu(II) content in the outer aqueous solution (e.g., 200 mM), while a more porous and loose structure was observed for microspheres fabricated with a lower Cu(II) content (e.g., 25 or 50mM) in the outer aqueous solution (Figure 9). Mercury intrusion porosimetry results in Figure 10 showed the pore size decreased from 2252 to 610 nm and the pore volume decreased from 3.54 to 1.28 mL/g while the Cu(II) content in the outer aqueous solution increased from 25 to 200 mM. A much faster cross-linking could be accomplished with a higher osmotic pressure in the high Cu(II) content solution, resulting in a much more compact structure of the resultant microspheres. Therefore, the pore size and pore volume decreased upon the increase of the Cu(II) content in the outer aqueous solution. Due to the same W1/O ratio in the fabrication process, the complexed Cu(II) amount of the microspheres were almost the same (≈2.6%).

### 3.3. Evaluation of CMC/CA Microspheres as Absorbents in Cigarette Smoke

In order to evaluate the adsorption performance of CMC/CA microsphere as filter additives, cigarettes with 15 mg/cig of CMC/CA microspheres and cigarettes with an empty cavity as blank sample were smoked under ISO smoking regimes. Smoke yields of HCN were analyzed and percentage reductions were calculated relative to the yields from the cigarette with empty cavity (Table 2).

With CMC/CA microspheres as absorbents, the HCN yield was 64.6–99.2 μg/cig, which was much lower than that of the control (129.5 μg/cig). The removal efficiency of HCN was about 23.4–50.1%. The higher removal efficiency was due to the high chemisorption capability of the microspheres for HCN. The loading cupric ions could complex with HCN and the connected porous structure enhanced the contact of HCN to the absorbs and thus enhanced the chemisorption capability of HCN from the cigarette smoke. 

The cupric loading amount was the most important factor that affected the chemisorption of HCN from cigarette smoke. Cu(II) was the active site to complex with HCN, and thus a large cupric loading amount resulted in a higher HCN removal efficiency. For example, the microsphere fabricated with higher W1/O ratio (e.g. 50/100–90/100) had a higher cupric loading amount, thus they had a higher HCN removal efficiency (e.g., 41–50%), while the microsphere fabricated with a lower W1/O (e.g., 10/100) had a relatively low HCN removal efficiency of 27%. The macrostructure also had great influence on the chemisorption of HCN from cigarette smoke due to the fact that cigarette smoke could pass through the connected macropores and achieve a fast chemisorption on the active site of Cu(II). For instance, the microspheres fabricated with different content of Cu(II) in the outer aqueous solution had a similar cupric loading amount of ≈2.6%, but the HCN removal efficiency varied from 23.4–50.1%. A much more compact microsphere structure, which resulted from a much higher content of Cu(II) in the outer aqueous solution, made it difficult for cigarette smoke to pass through the microspheres and adsorb on the active site. Compared to the functional ion-exchange resin [7], the CMC/CA composite microspheres had a considerable HCN removal efficiency but a less-applicable amount of only 15 mg per cigarette. In addition, the CMC/CA composite microspheres could be easily fabricated from two commercially available cellulose derivatives with a low cost/benefit ratio, showing great potential of these functional microspheres as HCN adsorbents in cigarette filter.

## 4. Conclusions

Porous carboxymethyl cellulose/cellulose acetate (CMC/CA) composite microspheres were prepared via the combination of emulsion–solvent evaporation and in situ ion cross-linking method. Cupric (II) ions, which have a high complexing ability toward HCN, were introduced to the CMC/CA microspheres during the in situ cross-linking process. These microspheres have a connected macroporous structure and a high cupric(II) loading amount of 0.8–4.1 wt %. By varying the preparation parameters, the porous structure and cupric(II) loading amount could be adjusted. Using these functional porous microspheres as adsorbents, the HCN content in cigarette smoke was reduced by up to 23.4–50.1%, showing the great potential of these functional microspheres in the chemisorption of HCN from cigarette smoke.

## Figures and Tables

**Table 1:** Preparation parameters and characterization of the microspheres

Sample	Preparation Parameters			Characterization of the Microspheres.			
	CMC (wt %)	W1/O (*v*/*v*)	Cu(II) in W2 (mM)	BET Surface Area (m^2^/g)	Pore Size (nm)	Pore Volume (mL/g)	The Cu(II) Loading Amount (wt %)
A-1	2	50:100	50	5.06	952	3.23	1.8
A-2	3	50:100	50	6.46	1780	2.97	2.6
A-3	4	50:100	50	5.75	2264	2.45	3.3
B-1	3	10:100	50	2.24	1058	1.04	0.8
B-2	3	30:100	50	3.87	540	2.07	1.8
B-3	3	50:100	50	6.46	1780	2.97	2.6
B-4	3	70:100	50	13.50	1037	5.69	3.4
B-5	3	90:100	50	7.42	1905	5.20	4.1
C-1	3	50:100	25	5.86	2252	3.54	2.7
C-2	3	50:100	50	6.46	1780	2.97	2.6
C-3	3	50:100	100	3.86	898	2.12	2.6
C-4	3	50:100	200	2.80	610	1.28	2.6

**Table 2:** The HCN removal efficiency of CMC/CA microspheres.

Sample	HCN Yield (μg/cig)	HCN Removal Efficiency (%)
control	129.5	/
A1	89.6	30.9
A2	64.6	50.1
A3	69.8	46.1
B1	94.1	27.3
B2	82.7	36.1
B3	64.6	50.1
B4	75.4	41.8
B5	71.5	44.8
C1	69.6	46.3
C2	64.6	50.1
C3	88.8	31.4
C4	99.2	23.4

## References

[1] Rodgman A., Perfetti T.A. (2008). The Chemical Components of Tobacco and Tobacco Smoke.

[2] WHO (2008). The Scientific Basis of Tobacco Product Regulation, Second Report of a WHO Study Group.

[3] Piade J.J., Wajrock S., Jaccard G., Janeke G. (2013). Formation of mainstream cigarette smoke constituents prioritized by the World Health Organization—Yield patterns observed in market surveys, clustering and inverse correlations. Food Chem. Toxicol..

[4] Branton P., Bradley R.H. (2010). Activated carbons for the adsorption of vapours from cigarette smoke. Adsorpt. Sci. Technol..

[5] Branton P.J., McAdam K.G., Duke M.G., Liu C., Curle M., Mola M., Proctor C.J., Bradley R.H. (2011). Use of classical adsorption theory to understand the dynamic filtration of volatile toxicants in cigarette smoke by active carbons. Adsorpt. Sci. Technol..

[6] Barnes P.A., Chinn M.J., Dawson E.A., Norman P.R. (2002). Preparation, characterisation and application of metal-doped carbons for hydrogen cyanide removal. Adsorpt. Sci. Technol..

[7] Branton P.J., McAdam K.G., Winter D.B., Liu C., Duke M.G., Proctor C.J. (2011). Reduction of aldehydes and hydrogen cyanide yields in mainstream cigarette smoke using an amine functionalised ion exchange resin. Chem. Cent. J..

[8] Branton P.J., Lu A.H., Schüth F. (2009). The effect of carbon pore structure on the adsorption of cigarette smoke vapour phase compounds. Carbon.

[9] He S., Zhang F., Cheng S., Wang W. (2016). Synthesis of sodium acrylate and acrylamide copolymer/GO hydrogels and their effective adsorption for Pb^2+^ and Cd^2+^. ACS Sustain. Chem. Eng..

[10] Gupta V.K., Agarwal S., Singh P., Pathania D. (2013). Acrylic acid grafted cellulosic Luffa cylindrical fiber for the removal of dye and metal ions. Carbohydr. Polym..

[11] Liu Y., Wang W., Wang A. (2010). Adsorption of lead ions from aqueous solution by using carboxymethyl cellulose-g-poly (acrylic acid)/attapulgite hydrogel composites. Desalination.

[12] Li D., Li Q., Mao D., Bai N., Dong H. (2017). A versatile bio-based material for efficiently removing toxic dyes, heavy metal ions and emulsified oil droplets from water simultaneously. Bioresour. Technol..

[13] Rustemeyer P. (2004). 5.2 CA filter tow for cigarette filters. Macromol. Symp..

[14] Sata H., Murayama M., Shimamoto S. (2004). 5.4 Properties and applications of cellulose triacetate film. Macromol. Symp..

[15] Liu C.X., Bai R.B. (2006). Adsorptive removal of copper ions with highly porous chitosan/cellulose acetate blend hollow fiber membranes. J. Membr. Sci..

[16] Tian Y., Wu M., Liu R., Li Y., Wang D., Tan J., Wu R., Huang Y. (2011). Electrospun membrane of cellulose acetate for heavy metal ion adsorption in water treatment. Carbohydr. Polym..

[17] Cai Z.X., Wu J., Du B.Q., Zhang H.B. (2018). Impact of distribution of carboxymethyl substituents in the stabilizer of carboxymethyl cellulose on the stability of acidified milk drinks. Food Hydrocoll..

[18] Lee J., Park S., Roh H., Oh S., Kim S., Kim M., Kim D., Park J. (2018). Preparation and characterization of superabsorbent polymers based on starch aldehydes and carboxymethyl cellulose. Polymers.

[19] Park J., An S.J., Jeong S.I., Gwon H.J., Lim Y.M., Nho Y.C. (2017). Chestnut honey impregnated carboxymethyl cellulose hydrogel for diabetic ulcer healing. Polymers.

[20] Rasoulzadeh M., Namazi H. (2017). Carboxymethyl cellulose/graphene oxide bionanocomposite hydrogel beads as anticancer drug carrier agent. Carbohydr. Polym..

[21] Yang J., Li J. (2018). Self-assembled cellulose materials for biomedicine: A review. Carbohydr. Polym..

[22] Gasemloo S., Khosravi M., Sohrabi M.R., Dastmalchi S., Gharbani P. (2019). Response surface methodology (RSM) modeling to improve removal of Cr (VI) ions from tannery wastewater using sulfated carboxymethyl cellulose nanofilter. J. Clean. Prod..

[23] Chen Y., Long Y., Li Q., Chen X., Xu X. (2019). Synthesis of high-performance sodium carboxymethyl cellulose-based adsorbent for effective removal of methylene blue and Pb (II). Int. J. Biol. Macromol..

[24] Nakamoto K. (2009). Infrared and Raman Spectra of Inorganic and Coordination Compounds, Part B, Applications in Coordination, Organometallic, and Bioinorganic Chemistry.

